# Hypertriglyceridemia and Other Plasma Lipid Profile Abnormalities among People Living with Diabetes Mellitus in Ethiopia: A Systematic Review and Meta-Analysis

**DOI:** 10.1155/2021/7389076

**Published:** 2021-05-10

**Authors:** Baye Dagnew, Yigizie Yeshaw, Demeke Geremew, Dessie Abebaw Angaw, Henok Dagne, Mekuriaw Alemayehu, Meseret Derbew Molla, Yonas Akalu

**Affiliations:** ^1^Department of Human Physiology, School of Medicine, College of Medicine and Health Sciences, University of Gondar, P. O. Box 196, Gondar, Ethiopia; ^2^Department of Epidemiology and Biostatistics, Institute of Public Health, College of Medicine and Health Sciences, University of Gondar, Gondar, Ethiopia; ^3^Department of Immunology, College of Medicine and Health Sciences, University of Gondar, Gondar, Ethiopia; ^4^Department of Environmental and Occupational Health and Safety, Institute of Public Health, College of Medicine and Health Sciences, University of Gondar, Gondar, Ethiopia; ^5^Department of Biochemistry, School of Medicine, College of Medicine and Health Sciences, University of Gondar, Gondar, Ethiopia

## Abstract

**Background:**

Dyslipidemia is one of the leading causes of cardiovascular complications in diabetes mellitus (DM) patients. Though it is a major public health problem in Ethiopia, there is no a nation-wide study to determine dyslipidemia among DM patients yet. Therefore, this systematic review and meta-analysis intended to estimate the prevalence of hypertriglyceridemia and other plasma lipid abnormalities among people living with DM in Ethiopia.

**Methods:**

We systematically searched PubMed, Google Scholar, African Journals Online, Hinari, and direct Google. Studies conducted until May 9, 2020, that reports the prevalence of dyslipidemia among people living with DM were included. The DerSimonian and Laird random-effects model was used to determine the pooled prevalence of lipid profile abnormalities. Heterogeneity was checked using the *I*^2^ statistic, whereas publication bias was tested by funnel plot and Egger's test. Besides, subgroup and sensitivity analyses were performed.

**Results:**

We used 18 primary studies, including 4961 participants living with DM, which met the eligibility criteria for the meta-analysis of hypertriglyceridemia. The estimate of hypertriglyceridemia (≥150 mg/dl) was 48.15% (95% CI: 38.15-58.15, *I*^2^ = 98.4%) after performing the main meta-analysis using the random-effects model. The subgroup analysis showed a higher pooled estimate of hypertriglyceridemia among T2DM (57.80% (95% CI: 50.50-65.10), *I*^2^ = 92.5%), studies that used probability sampling technique (59.09% (95% CI: 43.58-74.59), *I*^2^ = 98.6%, *p* < 0.001), and studies from primary data sources (51.43% (95% CI: 40.72-62.13), *I*^2^ = 98.0%, *p* < 0.001). Moreover, the estimated pooled prevalence of the total plasma cholesterol (TC ≥ 200 mg/dl) was 34.08% (95% CI: 28.41-39.75, *I*^2^ = 92.4%), LDL − C ≥ 100 mg/dl was 41.13% (95% CI: 27.15-55.11, I^2^ = 98.8%), and HDL ≤ 40 mg/dl for men and ≤ 50 mg/dl for women was 44.36% (95% CI: 31.82-56.90, *I*^2^ = 98.8%).

**Conclusions:**

The pooled prevalence of hypertriglyceridemia and other lipid abnormalities among DM patients was relatively high in Ethiopia. It strongly suggests the need to give maximal attention to the adherence of DM management to reduce the circulatory lipid profile abnormalities and subsequent complications. *Prospero Registration*. CRD42020182291.

## 1. Background

Dyslipidemia is a derangement of lipoprotein metabolism characterized by an increased level of serum triglycerides (TG), total cholesterol (TC), low-density lipoprotein cholesterol (LDL-C), and decreased level of high-density lipoprotein cholesterol (HDL-C) [1]. It is a risk factor for cardiovascular diseases (CVDs) [2, 3]. Acquired dyslipidemia is caused by sedentary lifestyle such as an unhealthy diet and chronic illnesses such as obesity, chronic kidney disease, hepatic diseases, and DM [4–6]. In return, dyslipidemia contributes to the occurrence of CVDs in DM patients [7].

Dyslipidemia in DM patients is largely attributed to both increased production and delayed catabolism of very-low-density lipoprotein (VLDL) secondary to resistance and a relative deficiency of insulin, a key hormone that regulates lipid metabolism [3, 8]. Physiologically, insulin reduces VLDL production by inhibiting the activation of hormone-sensitive lipase at adipose tissue and decreases the circulating nonesterified free fatty acids from adipocytes. Thus, the production of nascent VLDL and apoprotein B100 (Apo B100) is inhibited because of limited free fatty acids in the circulation [9]. Increased hepatic lipogenesis also increases liver cholesterol [10]. Besides, insulin activates lipoprotein lipase (LPL) which metabolizes circulatory triglyceride and VLDL to free fatty acid and glycerol and also increases the expression of LDL receptors to facilitate the uptake of VLDL remnants by the tissues [3]. In DM patients, low, absence, or insulin resistance results in a rise of triglyceride and VLDL. The normal inhibitory effect of insulin on hepatic Apo B100 production and triglyceride secretion is lost, and the secreted VLDL becomes higher than utilization [11]. Insulin resistance causes loss of potent stimulatory effect of insulin on LPL and contributes to the increment of triglyceride in the plasma [12, 13]. Stimulation of LDL clearance via increasing LDL-C receptor expression and activity by insulin is also lost because of insulin resistance [3].

Diabetes mellitus is associated with vascular problems such as peripheral arterial disease [14] and hypertension [15]. Hypertriglyceridemia combined with low HDL-C or high LDL-C in DM populations contributes to life-threatening vascular complications [8, 16, 17] including ischemic stroke [18, 19]. Besides the morbidity and mortality, hypertriglyceridemia increases the health care costs of people living with DM [20].

Evidences revealed that poor nutritional knowledge, inadequate dietary practice, low level of physical activity [21], obesity [21, 22], poor glycemic control [23], being hypertensive [21–23], old age [21, 22], low education status [21], and duration of DM [22, 23] were significant predictors of dyslipidemia among people living with DM.

Globally, dyslipidemia causes around 2.6 million deaths and 29.7 million disability-adjusted life years (DALYs) [24, 25]. The prevalence of dyslipidemia, among DM patients, is 67.1% in China [26], 86% in Bangladesh [27], 88.9% in Thailand [28], and 89% in India [29] whereas, in Ethiopia, hypertriglyceridemia ranges from 14.2% [30] to 72.5% [31]. Apt management is essential [32] which is expensive in resource-limited settings like Ethiopia. Hence, measuring the prevalence and directing this scarce resource towards its prevention is the best solution. There is no comprehensive study conducted in Ethiopia to determine triglyceridemia and other plasma lipid profile abnormalities among people living with DM. Therefore, this meta-analysis intended to determine the pooled prevalence of dyslipidemia (high TG, low HDL-C, high LDL-C, and high TC) among people living with DM in Ethiopia.

## 2. Methods

### 2.1. Reporting and Registration

We conducted this systematic review and meta-analysis based on the principles of the Centre for Reviews and Dissemination's (CRD) guidance for undertaking reviews in health care [33], and the reporting system was following the Preferred Reporting Items for Systematic Review and Meta-Analysis (PRISMA) [34]. It is registered at Prospero with an identification number CRD42020182291 available at https://www.crd.york.ac.uk/prospero/#myprospero.

### 2.2. Data Sources

Major electronic databases (PubMed, Hinari, African Journals Online, and Google Scholar) were used to access relevant studies. Besides, a direct Google search was used to repossess a few articles after looking at the reference lists of included studies to account for the omission of the studies during searching of electronic databases. To account for unpublished data, we used the institutional repository of Addis Ababa University.

### 2.3. Searching Strategy

All pocket/primary studies reporting proportion or prevalence of hypertriglyceridemia and/or other plasma lipid abnormalities (abnormally high TC, high LDL-C, and low HDL-C), among people living with either T1DM or T2DM in Ethiopia, were the targets for this review. After identifying entry terms using the MeSH (medical subject heading) browser, relevant terms or phrases were combined using Boolean operators “OR” and “AND” to fit the advanced search by considering the CoCoPo (condition, context, population) principle. We used (“Dyslipidemia” [Title/Abstract] OR “Metabolic syndrome” [Title/Abstract]) OR “Dyslipidaemia” [Title/Abstract]) OR “Dyslipoproteinemias” [Title/Abstract]) OR “Hyperlipidemia” [Title/Abstract]) OR “high total Cholesterol” [Title/Abstract]) OR “high Triglycerides” [Title/Abstract]) OR “low ‘High Density Lipoprotein Cholesterol'” [Title/Abstract]) OR “elevated Low Density Lipoprotein Cholesterol'” [Title/Abstract]) AND “Diabetes mellitus” [Title/Abstract]) OR “Hyperglycemia” [Title/Abstract]) OR “Diabetes” [Title/Abstract]) AND “Ethiopia” [Title/Abstract] for searching articles in PubMed. We also used advanced searches for other databases.

### 2.4. Eligibility Criteria

#### 2.4.1. Inclusion Criteria

*Population*: people living with either T1DM or T2DM.

*Setting*: pocket studies conducted in Ethiopia or any localities of the country.

*The outcome of interest*: hypertriglyceridemia and other plasma lipid abnormalities (dyslipidemia) among people living with DM.

*Study design types*: observational studies (cross-sectional or case-control studies) reporting the prevalence or proportion of people with abnormal plasma lipid concentration.

*Publication status*: both published and unpublished studies were included.

*Publication language*: English.

*Publication date*: published until 9 May 2020.

### 2.5. Exclusion Criteria

Case-reports, case-series, letters to the journal editors, communications, and studies conducted on specific population were excluded.

### 2.6. Study Selection

Three independent authors (BD, DG, and YY) identified articles using electronic databases and other sources. All studies were exported from data sources into Endnote X7 for duplicate removal and citation. Two reviewers (BD and YY) selected relevant studies after evaluating title, abstract, and then full-text review for the abstraction of the data by using inclusion criteria. These reviewers harmonized most disagreements after discussion, and the third reviewer (DG) took part to resolve a few divergences between the two reviewers (BD and YY).

### 2.7. Quality Assessment of Included Studies

Two authors (HD and YA) investigated the quality of included studies using a modified Hoy et al. quality assessment tool [35]. This tool has 9 risks of bias items (each comprises zero or one risk of bias points) which have a maximum score of “9” and a minimum score of “0.” Ranking of risk of bias is labelled as low risk (0-3), moderate risk (4–6), and finally high risk (7–9). The third reviewer (MA) synchronized differences in quality appraisal between the two authors (HD and YA).

### 2.8. Data Abstraction

Structured data extraction sheet was prepared by two authors (HD and DAA) using Microsoft Excel 2010. The template of data extraction format contained the name of the primary author, publication year of the study, study design, regional state of the study, sample size, actual number of cases with specific plasma lipid abnormality, and percentage of individuals with specific lipid abnormality. Then, two authors (BD and YY) extracted the relevant data independently, and the extracted data of the two authors (BD and YY) were compared. Discrepancies were resolved by consensus after discussion.

### 2.9. Outcome Measurement

We used hypertriglyceridemia and other plasma lipid abnormalities as outcome variables. In the primary studies, the outcome variables were obtained by biochemical tests after drawing blood from each participant. Dyslipidemia is defined based on National Cholesterol Education Program Adult Treatment Panel III guideline which comprised either single or a combination of plasma lipid abnormalities. The following criteria were used for the diagnosis of overall dyslipidemia, i.e., TC ≥ 200 mg/dl, HDL − c < 40 mg/dl for males and < 50 mg/dl for females, LDL − c ≥ 100 mg/dl, and TG ≥ 150 mg/dl [36].

### 2.10. Statistical Analysis

Endnote X7 and Stata 11 were used for bibliographical management and statistical analysis, respectively. Heterogeneity was tested using the *Q* statistic, and *I*^2^ test was used to identify possible interstudy variations. The *I*^2^ values 25%, 50%, and 75% denote low, moderate, and high heterogeneity, respectively [37]. We used the DerSimonian and Laird random-effects model [38] for the main meta-analysis of the pooled estimates of the prevalence of hypertriglyceridemia and other plasma lipid abnormalities. Subgroup analysis was considered to account for possible heterogeneity. Publication bias (using the Funnel plot and Egger's test) [39], trim and fill [40], and sensitivity analyses were performed. For each plasma lipid abnormality, the point estimate was shown by the forest plot.

## 3. Results

### 3.1. The Review Process and Characteristics of the Included Studies

**Figure 1:**
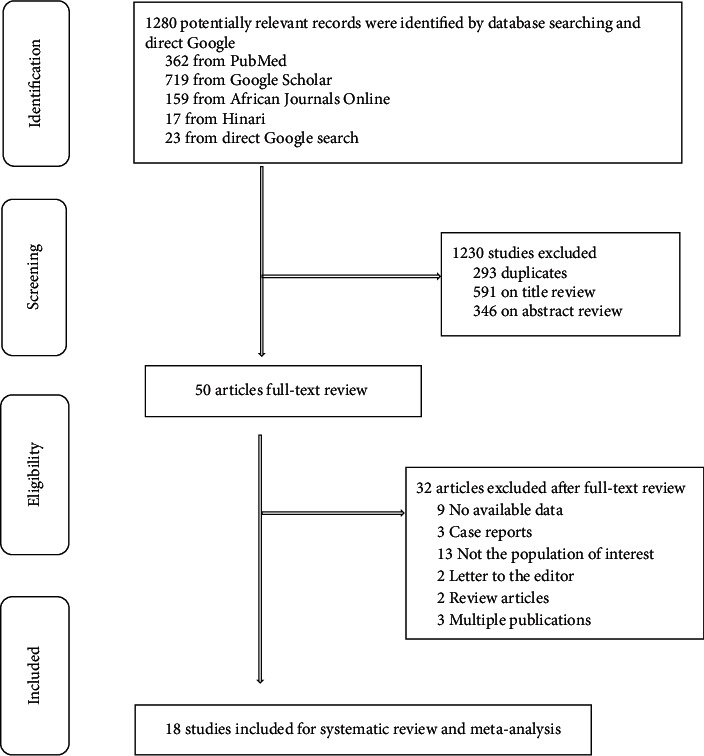
A PRISMA flow chart illustrating study selection process included for systematic review and meta-analysis of hypertriglyceridemia among people living with diabetes mellitus.

Eighteen primary studies (with 4961 participants living with either T1DM or T2DM) [22, 30, 31, 41–55] that met the inclusion criteria for the meta-analysis of hypertriglyceridemia were analyzed (Figure 1). Sixteen studies [22, 30, 31, 41–45, 47–51, 53–55] were published from 2003 to 2019, and only two studies were unpublished [46, 52]. A sample size of included studies ranged from 77 [47] to 581 [48]. Most studies were conducted in Addis Ababa [30, 43, 46, 48, 50, 52], and one study in Oromia region [47] (Table 1).

### 3.2. The Pooled Prevalence of Hypertriglyceridemia among People Living with DM

**Figure 2:**
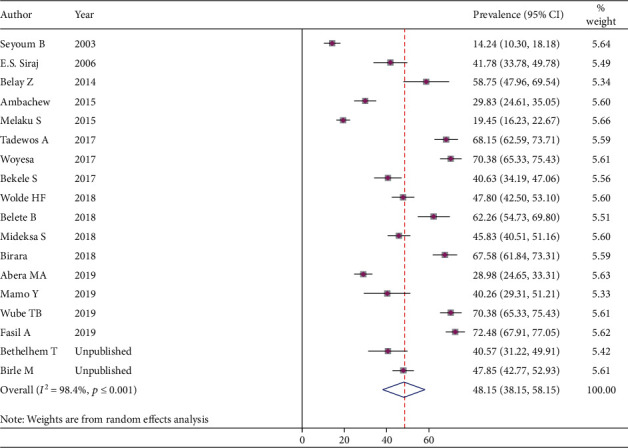
The pooled prevalence of hypertriglyceridemia among DM in Ethiopia.

In the fixed-effects model analysis, the pooled prevalence of hypertriglyceridemia was 43.9% (95% CI: 42.65-45.16%, prevalent cases = 2298) with substantial interstudy variation (heterogeneity). To account for this, we performed the main meta-analysis using the DerSimonian and Laird random-effects model to determine the pooled prevalence of hypertriglyceridemia, and it was 48.15% (95% CI: 38.15-58.15) (Figure 2).

### 3.3. Other Dyslipidemia among People Living with DM in Ethiopia

**Figure 3:**
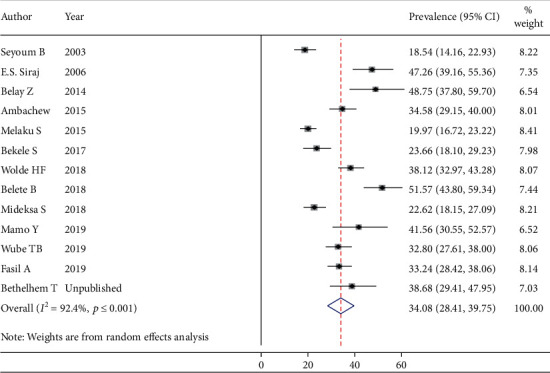
The pooled prevalence of total cholesterolemia among DM patients.

**Figure 4:**
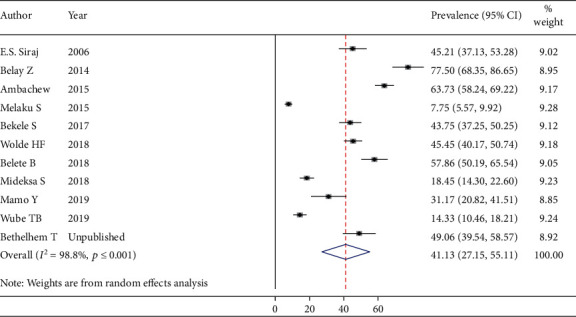
Estimated pooled prevalence of high “LDL_C” among DM patients.

**Figure 5:**
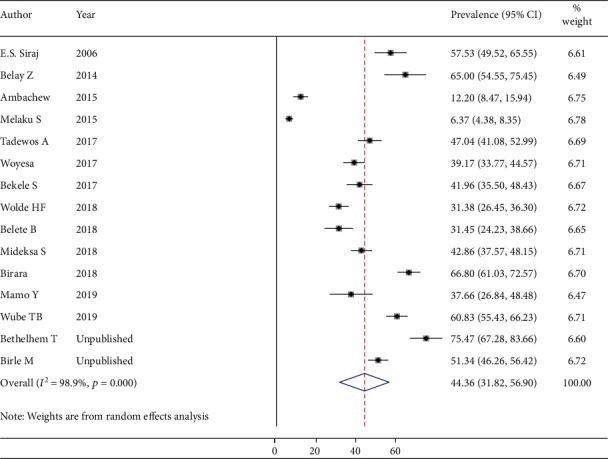
Forest plot to show pooled prevalence of low “HDL_C” among D patients.

Thirteen studies (3328 participants) were eligible for the estimation of the prevalence of total cholesterolemia among people living with DM. After running the main meta-analysis using the random-effects model, the pooled prevalence of high TC was 34.08% (prevalent cases = 1021) (Figure 3). Eleven primary studies (2659 participants) were included for the meta-analysis of elevated “LDL-C” with the prevalence of 41.13% (prevalent cases = 889) (Figure 4). Finally, fifteen studies (3871 participants) were eligible for meta-analysis of low “HDL-C,” and the pooled prevalence was 44.36% (prevalent cases = 1432) (Figure 5).

### 3.4. Subgroup Analysis of the Pooled Prevalence of Hypertriglyceridemia

As one of the handling mechanisms of heterogeneity, we performed subgroup analysis for the pooled prevalence of hypertriglyceridemia by the type of DM, study design, sampling technique, data source, and publication status. Random-effects analysis showed a 57.80% (9% CI: 50.50-65.10) prevalence of hypertriglyceridemia among T2DM patients whereas 48.64% of the pooled prevalence was observed among published studies (Table 2).

### 3.5. Metaregression

We applied metaregression to identify the source of heterogeneity. Metaregression was performed using a type of DM used as population interest, sampling procedure, data source, study design, and publication status of the included studies. Of these, only a type of DM was picked as a source of heterogeneity (*p* ≤ 0.05) (Table 3).

### 3.6. Publication Bias

**Figure 6:**
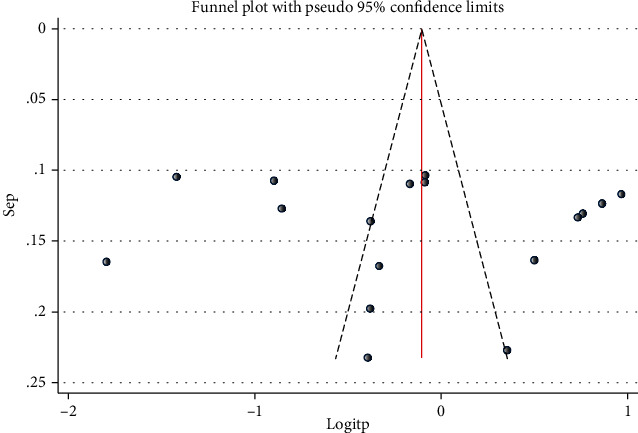
Funnel plot to show publication bias for triglyceridemia.

As indicated by the funnel plot (Figure 6), there was an asymmetrical distribution of studies which is an evidence for publication bias. However, when we performed objective publication bias (egger) test, the bias coefficient of Egger's test was 1.255 (*p* > 0.05) that indicates absence of publication bias in our study.

### 3.7. Sensitivity Analysis

The leave-one-out method was used to identify a significant single study effect on the combined estimate. As shown in Table 4, no study exerted a significant effect on the overall pooled prevalence of hypertriglyceridemia among people living with DM.

## 4. Discussion

Dyslipidemia is one of the leading causes of cardiovascular diseases [56]. Given its public health significance, scholars conducted many pocket studies in different populations. However, there is no comprehensive study in Ethiopia. The main aim of the current systematic review and meta-analysis was to describe the pooled prevalence of plasma lipid abnormalities (TG, TC, LDL-C, and HDL-C) among people living with DM in Ethiopia. Accordingly, the pooled prevalence was 48.15% for TG, 34.08% for TC, 41.13% for LDL-C, and 44.36% for HDL-C. This indicates a presence of a huge number of individuals with dyslipidemia in Ethiopia, which necessitates the health sector stakeholders to design and implement preventive strategies for plasma lipid abnormalities and hence minimizing dyslipidemia-associated complications of DM and improving their quality of life.

In this meta-analysis, we found a substantial interstudy variation anticipated to affect the pooled prevalence of hypertriglyceridemia and other plasma lipid abnormalities. For the sake of handling the heterogeneity, we used random-effects model analysis for the final combined prevalence of each outcome variable. Even after running the main meta-analysis using the random-effects analysis, there was heterogeneity. Besides, subgroup analysis was executed using type of DM, study design, sampling technique, data source, and publication status.

The prevalence of hypertriglyceridemia among T2DM was 57.8% which is similar with studies in Tanzania (53.8%) [57] and India (56.1%) [58] but lower than that in a finding in Jordan (83.1%) [59]. The pooled prevalence was 48.64% among the published studies which is similar with the overall pooled prevalence of the current study (48.15%). Furthermore, metaregression was performed to sort out the real cause of heterogeneity from which the type of DM was significant source of heterogeneity.

In the current study, the pooled prevalence of hypertriglyceridemia among DM patients was 48.15%. This higher percentage could be related with the defects of insulin action [60, 61], in that low insulin or insulin resistance leads to reduced fatty acid mobilization into cells and increases lipolysis, both of which contributed to high plasma lipid levels [62]. Other studies in in Thailand (49.94%) [28], Botswana (38.9%) [63], Tanzania (53.8%) [57], India (56.1%) [58], Brazil (46.7%) [64], and Yemen (39.2%) [65] also reported similar findings. However, the pooled prevalence of hypertriglyceridemia, in this study, is lower than a finding of the local study in Jordan (83.1%) [59]. This difference might be due to the variations in the study setting and population characteristics. In contrary to this, our study finding is higher than other reports in the United States (30%) [66], Saudi Arabia (17%) [67], China (22.3%) [68], and Korea (28.7%) [69]. The reason for this might emanate from the differences in sample size, treatment options, adherence to self-care, and duration of DM.

In this review, the pooled prevalence of plasma TC was 34.08% which is in line with findings in Botswana (33.5%) [63] and India (36.3%) [58]. However, it is lower than a country-based study in Thailand (35.05%) [28] and local studies in Tanzania (49.6%) [57], Yemen (52.5%) [65], and Jordan (77.2%) [59]. This disagreement might be explained by the differences in the study population in which our study included both type 1 and 2 DM patients, whereas other studies included only people living with T2DM. It is known that people living with T2DM are more prone to plasma lipid abnormalities than T1DM [70]. It can also be due to the differences in the duration of DM, sample size, and treatment adherence. However, the current estimate of TC is higher than nation-wide studies in China [68], another study in China (6.9%) [71], Saudi Arabia (13.8%) [67], and Korea (14.5%) [69]. This might be due to lifestyle and socioeconomic differences between the study populations.

The pooled prevalence of high LDL-C was 41.13%, which is akin to an evidence in the United States (53%) [66]. However, LDL-C in our study is lower than other studies in Thailand (56.54%) [28], Tanzania (72.3%) [57], India (57.3%) [58], Brazil (79%) [64], Yemen (67.5%) [65], and Jordan (91.5%) [59]. The reason for the lower estimated high LDL-C in our review could be due to variations in sample size and scope of the study. However, the estimate of elevated LDL-C is higher than that of other studies in China [68], Saudi Arabia (12.85) [67], and Korea (14.8%) [69]. This could be due to variations in the number of participants, self-management, and duration of DM.

Finally, we estimated the prevalence of abnormally low HDL-C (44.36%) among people living with DM which is in line with other nation-wide studies in Saudi Arabia (40%) [67], Korea (41.2%) [69], and China (40.8%) [68] and local studies in India (35.7%) [58] and Brazil (34.9%) [64]. The estimate of low HDL-C is higher than studies in the United States (23%) [66] and Yemen (25.5%) [65]. This might be due to differences in the knowledge level of participants, self-care practice, and case definition used in each study. Participants in the United States are more likely to have better self-care practice. Our review finding is lower than studies conducted in Thailand (59.59%) [28], Jordan (83.9%) [59], and Tanzania (63%) [57]. This difference could be due to variations in the study population, socioeconomic status, and duration of DM.

As stated, this meta-analysis focused on pooling the prevalence of hypertriglyceridemia and other lipid abnormalities due to lack of studies which reported associated factors of dyslipidemia. In other studies, dyslipidemia is associated with increased age, urbanization, female sex, higher glucose level, smoking, and hypertension [72, 73], all of which are modifiable. The findings of this review suggest a need for comprehensive strategies to prevent plasma lipid abnormalities so that dyslipidemia-associated metabolic complications of DM can be avoided by focusing on modifiable risk factors such as lifestyle changes to achieve standard targets. In addition to this, we recommend researchers to conduct a study on determinant factors of dyslipidemia among DM patients so that strategies will be built by targeting modifiable risk factors.

### 4.1. Limitations of the Study

This review was the first in its kind on this topic and conducted with a robust contribution of different disciplines. However, the finding of this study should be interpreted with the following limitations. Firstly, the total participants included were not a representative of the national figure which might be difficult to generalize. But, it can be used as a good finding than other primary studies since it was a pooled prevalence of others. Secondly, there was high heterogeneity. We performed the random-effects, subgroup analysis, and metaregression to chase for the real cause of the variation, and a type of DM was the cause for heterogeneity. Thirdly, there was lack of studies in certain regions of the country and each year which might be difficult to use the results as a baseline in those regions. Finally, the included studies lack associated factors of dyslipidemia that impeded us to analyze the pooled effect of individual factors on plasma lipid abnormalities.

## 5. Conclusions

Our systematic review and meta-analysis revealed a high estimated prevalence of plasma lipid abnormalities, particularly TG among people living with DM. Patients need to adhere to both nonpharmacologic and pharmacologic therapies to reduce abnormal plasma lipid levels and prevent dyslipidemia-associated health risks of DM. Opportunistic test strategies to dyslipidemia parameters have to be strictly followed by all people living with DM at follow-up clinics.

## Figures and Tables

**Table 1:** Characteristics of included studies for the meta-analysis of hypertriglyceridemia and other plasma lipid abnormalities among people living with diabetes mellitus in Ethiopia.

Author	Year	Study region	Study design	Type of DM	Sample size	TC ≥ 200 mg/dl (*n*)	TC ≥ 200 mg/dl (%)	TG ≥ 150 mg/dl (*n*)	TG ≥ 150 mg/dl (%)	LDL − C ≥ 100 mg/dl (*n*)	LDL − C ≥ 100 mg/dl (%)	HDL − C < 40 mg/dl (*n*)	HDL − C < 40 mg/dl (%)	Quality status
Seyoum B [30]	2003	Addis Ababa	CS	Mixed	302	56	18.5	43	14.2	—	—	—	—	Low risk
E.S. Siraj [50]	2006	Addis Ababa	CS	Mixed	146	69	47.3	61	41.8	66	45.2	84	57.5	Low risk
Belay Z [43]	2014	Addis Ababa	CC	T2DM	80	39	48.8	47	58.8	62	77.5	52	65.0	Low risk
Melaku S [48]	2015	Addis Ababa	CS	Mixed	581	116	20.0	113	19.4	45	7.7	37	6.4	Low risk
Ambachew [42]	2015	SNNP	CS	Mixed	295	102	34.6	88	29.8	188	63.7	36	12.2	Low risk
Bekele S [22]	2017	SNNP	CS	Mixed	224	53	23.7	91	40.6	98	43.8	94	42.0	Low risk
Tadewos A [51]	2017	SNNP	CS	T2DM	270	—	—	184	68.1	—	—	127	47.0	Low risk
Woyesa [54]	2017	SNNP	CS	T2DM	314	—	—	221	70.4	—	—	123	39.2	Low risk
Mideksa S [49]	2018	Tigray	CS	Mixed	336	76	22.6	154	45.8	62	18.5	144	42.9	Low risk
Belete B [44]	2018	Amhara	CS	T2DM	159	82	51.6	99	62.3	92	57.9	50	31.4	Low risk
Birara [45]	2018	Amhara	CS	T2DM	256	—	—	173	67.6	—	—	171	66.8	Low risk
Wolde HF [53]	2018	Amhara	CS	T2DM	341	130	38.1	163	47.8	155	45.5	107	31.4	Low risk
Abera MA [41]	2019	Tigray	CS	Mixed	421	—	—	122	29.0	—	—	—	—	Low risk
Mamo Y [47]	2019	Oromia	CC	T2DM	77	32	41.6	31	40.3	24	31.2	29	37.7	Low risk
Fasil A [31]	2019	Amhara	CS	Mixed	367	122	33.2	266	72.5	—	—	—	—	Low risk
Wube TB [55]	2019	SNNP	CS	T2DM	314	103	32.8	221	70.4	45	14.3	191	60.8	Low risk
Bethelhem T [52]	2015^∗^	Addis Ababa	CS	T2DM	106	41	38.7	43	40.6	52	49.1	80	75.5	Low risk
Birle M [46]	2019^∗^	Addis Ababa	CS	T2DM	372	—	—	178	47.8	—	—	191	51.3	Low risk

^∗^Studies not published, but the showed year depicts the study period. CS: cross-sectional; CC: case-control.

**Table 2:** Subgroup analysis of hypertriglyceridemia among people living with DM.

Subgroup analysis by	Characteristics	Degree of freedom	D+L pooled estimate with 95% CI	*I* ^2^ (*p* value)
Type of DM	T2DM	9	57.80 (50.50-65.10)	92.5% (*p* ≤ 0.001)
	Mixed	7	36.60 (22.79-50.41)	98.5% (*p* ≤ 0.001)
Publication status	Published	15	48.64 (37.55-59.72)	98.6% (*p* ≤ 0.001)
	Unpublished	1	45.31 (38.51-52.11)	44.5% (*p* > 0.05)
Study design	Case-control	1	48.15 (38.15-58.15)	82.0% (*p* ≤ 0.01)
	Cross-sectional	15	47.99 (37.27-58.71)	98.6% (*p* ≤ 0.001)
Sampling technique	Probability	8	59.09 (43.58-74.59)	98.6% (*p* ≤ 0.001)
	Nonprobability	6	37.80 (27.82-47.79)	95.6% (*p* ≤ 0.001)
	Unknown	1	34.00 (22.75-45.26)	79.4% (*p* ≤ 0.05)
Data source	Primary	14	51.43 (40.72-62.13)	98.0% (*p* ≤ 0.001)
	Secondary	2	31.96 (16.50-47.41)	97.5% (*p* ≤ 0.001)

**Table 3:** Metaregression analysis to identify a possible source of heterogeneity in the meta-analysis of hypertriglyceridemia.

Logitp	Coef.	Std. Err.	*T*	*p* > *t*	95% CI	
					Lower limit	Upper limit
Data source	-.8223736	.4131817	-1.99	0.070	-1.722619	.077872
Sampling procedure	.1127625	.2499427	0.45	0.660	-.4318159	.6573409
Study design	.707935	.533111	1.33	0.209	-.4536141	1.869484
Type of DM	1.086432	.3585828	3.03	0.010	.3051472	1.867717
Publication status	-.9690825	.5086497	-1.91	0.081	-2.077335	.13917
_cons	-1.278057	1.297495	-0.99	0.344	-4.105056	1.548942

**Table 4:** Leave-one-out sensitivity analysis for the pooled prevalence of hypertriglyceridemia among people living with DM.

Study omitted	Estimate	(95% CI)	
		Lower limit	Upper limit
Seyoum B (2003)	50.18	40.62	59.74
E.S. Siraj (2006)	48.52	38.09	58.96
Belay Z (2014)	47.55	37.23	57.88
Melaku S (2015)	49.87	40.20	59.55
Ambachew (2015)	49.24	38.75	59.73
Bekele S (2017)	48.59	38.08	59.11
Tadewos A (2017)	46.97	36.76	57.17
Woyesa (2017)	46.83	36.75	56.91
Mideksa S (2018)	48.29	37.67	58.91
Belete B (2018)	47.33	36.99	57.67
Birara (2018)	47.00	36.77	57.23
Wolde HF (2018)	48.17	37.56	58.79
Abera MA (2019)	49.29	38.77	59.83
Mamo Y (2019)	48.59	38.24	58.96
Fasil A (2019)	46.69	36.82	56.58
Wube TB (2019)	46.83	36.75	56.91
Bethelhem T (2015^∗^)	48.59	38.19	58.98
Birle M (2019^∗^)	48.17	37.53	58.81
Combined	48.15	38.15	58.15

^∗^Studies not published, but the indicated year depicts the study conduction period.

## Data Availability

Dataset can be accessed from the corresponding author upon reasonable request.

## Abbreviations

CI: Confidence interval
HDL-C: High-density lipoprotein cholesterol
LDL-C: Low-density lipoprotein cholesterol
TC: Total cholesterol
T1DM: Type 1 diabetes mellitus
T2DM: Type 2 diabetes mellitus.
